# Catalytic robustness and torque generation of the F_1_-ATPase

**DOI:** 10.1007/s12551-017-0262-x

**Published:** 2017-03-25

**Authors:** Hiroyuki Noji, Hiroshi Ueno, Duncan G. G. McMillan

**Affiliations:** Department of Applied Chemistry, Graduate School of Engineering, The University of Tokyo, Tokyo, 113-8656 Japan

**Keywords:** F_1_-ATPase, ATP synthase, Single-molecule techniques, Molecular motor

## Abstract

The F_1_-ATPase is the catalytic portion of the F_o_F_1_ ATP synthase and acts as a rotary molecular motor when it hydrolyzes ATP. Two decades have passed since the single-molecule rotation assay of F_1_-ATPase was established. Although several fundamental issues remain elusive, basic properties of F-type ATPases as motor proteins have been well characterized, and a large part of the reaction scheme has been revealed by the combination of extensive structural, biochemical, biophysical, and theoretical studies. This review is intended to provide a concise summary of the fundamental features of F_1_-ATPases, by use of the well-described model F_1_ from the thermophilic *Bacillus* PS3 (TF_1_). In the last part of this review, we focus on the robustness of the rotary catalysis of F_1_-ATPase to provide a perspective on the re-designing of novel molecular machines.

## Introduction

The F_o_F_1_ ATP synthase is a ubiquitous enzyme, spreading across all kingdoms of biology. It is found in the inner membrane of mitochondria, the thylakoid membrane of chloroplasts, and the plasma membranes of bacteria and some archaea. However, most archaea, and some eukaryotic bacteria, employ the evolutionary related V-type ATPase as an ATP synthase (Yoshida et al. 2001). F_o_F_1_ ATP synthases catalyze ATP synthesis using ADP and inorganic phosphate as substrates. ATP synthesis is a thermodynamically uphill reaction. To catalyze ATP synthesis, the F_o_F_1_ ATP synthase utilizes an electrochemical potential of protons or sodium ions across biological membranes (proton motive force, *pmf*, or sodium motive force, *smf*) as the driving force (Abrahams et al. 1994; Dimroth et al. 2006; Yoshida et al. 2001). The use of chemiosmotic catalysis by the ATP synthase is such a prevalent reaction in biology that the F_o_F_1_ is widely considered to be one of the most ancient enzymes. A scenario on the last universal common ancestor (LUCA) proposes that an ancestor molecular complex of ATP synthase prebiotically emerged to confer energy production for LUCA (Lane and Martin 2012), although there are arguments against this scenario (Jackson 2016).

### F_o_ and F_1_

**Fig. 1:**
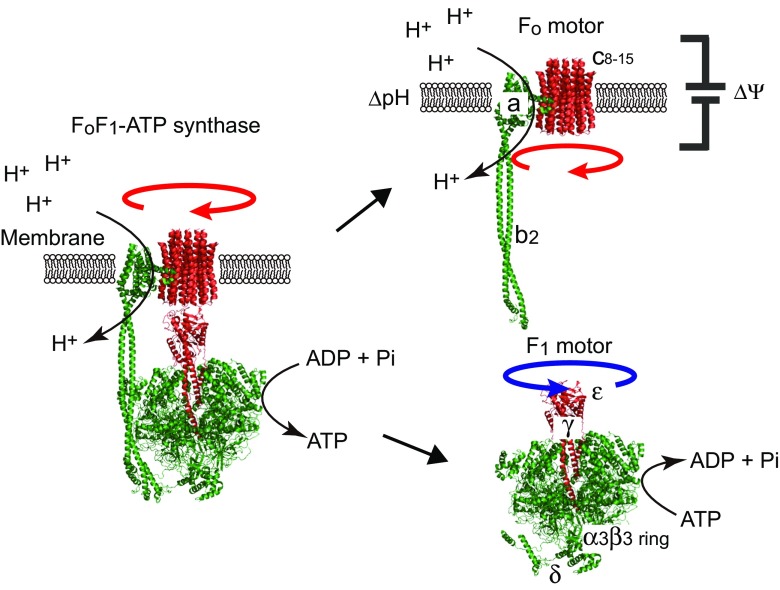
Two rotary motors of F_o_F_1_-ATP synthase. Schematic models of the F_o_F_1_-ATP synthase. The structural models of F_o_F_1_ (PDB ID: 5T4O) (Sobti et al. 2016) are shown as cartoon representation. The rotor and stator parts are shown in red and green, respectively. The F_o_F_1_-ATP synthase is composed of two tethered rotary motors, F_o_ and F_1_, each driven by two different fuels. The subunit composition of bacterial F_1_ and F_o_ are α_3_β_3_γδε and *ab*_2_ *c*_x_ (where x is the copy number of *c* subunits, which varies from 8 to 15 in different species), respectively. The membrane-embedded F_o_ motor rotates the *c*-ring (rotor) against the *ab*_2_ (stator), clockwise when viewed from the membrane side, which is driven by *pmf* consisting of membrane potential (ΔΨ) and proton concentration gradient (ΔpH). The F_1_ is an ATP-driven rotary motor in which the γ subunit (rotor) rotates against the α_3_β_3_-ring (stator). The ε subunit binds to the protruding part of the γ subunit. The δ binds to the bottom of the α_3_β_3_-ring. Note that the rotational direction of F_1_ is opposite to that of F_o_. In the whole complex of F_o_F_1_, F_o_ reverses the rotation of F_1_, leading to ATP synthesis from ADP and Pi

The F_o_F_1_ ATP synthase is a multisubunit membrane-integrated enzyme with a molecular weight of >500 kDa. This enzyme is composed of two structurally and functionally distinct portions, F_o_ and F_1_, each of which can be considered to be a rotary molecular motor (Junge et al. 1997; Noji and Yoshida 2001; Oster and Wang 2000). The F_o_ (∼120 kD) is the membrane-embedded portion of ATP synthase (Fig. 1). Bacterial F_o_ have the simplest subunit composition, *a*_1_ *b*_2_ *c*_x_, where the number of *c* subunits varies from 8 to 15 among different organisms, yet remains invariant within individual organisms (Ballhausen et al. 2009; Matthies et al. 2009; Meier et al. 2005b; Pogoryelov et al. 2007; Stock et al. 1999). F_o_ mediates proton or sodium ion translocation across the membrane and, in doing so, the *c* oligomer ring rotates against the *ab*_2_ stator complex. The *a* subunit is comprised of two half-channels, one exposed to each side of the membrane. Protons/sodium ions enter through one half-channel (entry channel) and are delivered to the *c* oligomer. Upon one 360° rotation of the *c* oligomer, each proton/sodium ion is redelivered to the *a* subunits through the other half-channel (exit channel) and released into the cell (Allegretti et al. 2015; Junge et al. 1997). Due to the difficulties with purification, handling, and analysis of the membrane-embedded F_o_, the functional and structural analyses of the F_o_ has long lagged behind the understanding we have of the F_1_. Recently, the structural information of how the *a* subunit forms the proton translocation path together with the *c* subunit has been available (Allegretti et al. 2015). However, together with crystallographic analysis, the recent advances in single-particle analysis together with electron microscopy are rapidly advancing our understanding of the structural detail of F_o_ (Morales-Rios et al. 2015). However, this review is not focused on the latest functional and structural studies on F_o_. Readers are encouraged to visit other reviews that more than adequately address this (Junge and Nelson 2005; Kühlbrandt and Davies 2016).

### F_1_-ATPase

**Fig. 2:**
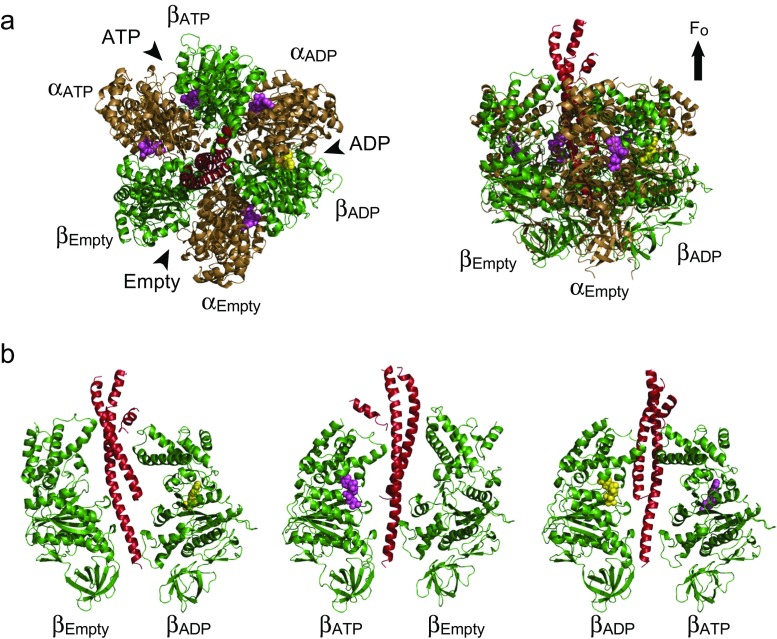
Crystal structures of F_1_. The crystal structures of F_1_ from bovine mitochondria (PDB ID: 1BMF) (Abrahams et al. 1994) are shown in cartoon diagrams as a top view from the membrane side (**a**, left) and as a side view (**a**, right and **b**). **a** The α, β, and γ subunits are shown in dark yellow, green, and red, respectively. The bound AMP-PNP and ADP are shown in magenta and yellow, respectively. The catalytic sites are located at the interfaces between α and β subunits (*black arrowheads*), which are mainly harbored by the β subunits. Each carries AMP-PNP, ADP, or none. Therefore, each β subunit catalytic site at any one point in time is designated as β_ATP_, β_ADP_, or β_Empty_, respectively. The non-catalytic sites are located at the other interfaces, all of which are occupied with AMP-PNP. Each α subunit forming a catalytic site is designated as α_ATP_, α_ADP_, or α_Empty_, respectively. The protruding part of the γ subunit is directed toward the F_o_ side. **b** Three β–β pairs with different nucleotide states are shown with the γ subunit. Both α and β subunits are composed of the N-terminal domain, nucleotide-binding domain, and C-terminal domain (from bottom to top). β_Empty_ takes an open conformation, and both β_ATP_ and β_ADP_ take a closed conformation with bound nucleotide. The C-terminal domain of the closed β subunit appears to push the γ subunit

The F_1_ is the water-soluble and catalytic portion of the F_o_F_1_ ATP synthase. The subunit composition of bacterial F_1_ is α_3_β_3_γ_1_δ_1_ε_1_. The F_1_ domain of most described enzymes show high ATP hydrolytic activity, typically in the range of 10^2^–10^3^ turnover/s (Bilyard et al. 2013; McMillan et al. 2016; Spetzler et al. 2006; Yasuda et al. 2001). For this reason, the F_1_ is frequently referred to as the ‘F_1_-ATPase’. However, the ATP hydrolytic activities of some the F_1_ are latent and inhibited in various ways (Cingolani and Duncan 2011; Keis et al. 2006; McMillan et al. 2007; Morales-Rios et al. 2015). This suppressive regulation is thought to avoid the futile consumption of ATP in a cell (Feniouk and Yoshida 2008). The minimum complex of F_1_ as a rotary molecular motor is the α_3_β_3_γ_1_ subcomplex, in which the γ subunit is inserted in the central cavity of the α_3_β_3_-ring. Crystal structures of F_1_ have revealed fundamental aspects of F_1_ (Fig. 2; Abrahams et al. 1994). The catalytic reaction centers of F_1_ for ATP hydrolysis/synthesis are located at the αβ interfaces (Weber and Senior 1997). Most of the amino acid residues that form the ATP-binding pocket reside in the β subunit, while the α subunit possesses one catalytically critical arginine residue (Ahmad and Senior 2005; Hayashi et al. 2012; Kagawa et al. 2004; Komoriya et al. 2012).

Upon ATP hydrolysis, the β subunit undergoes a large conformational change to induce the unidirectional rotation of the γ subunit, counterclockwise when viewed from the membrane side (Abrahams et al. 1994; Masaike et al. 2008; Noji et al. 1997). The ε subunit binds to the protruding domain of the γ to form the binding interface to F_o_. The ε subunit is also known to be a catalytic regulator (Cingolani and Duncan 2011; Feniouk et al. 2010; Hara et al. 2001; Keis et al. 2006; Tsunoda et al. 2001). The δ subunit attaches to the bottom tip of the α_3_β_3_-ring (Dunn and Chandler 1998; Ogilvie et al. 1997).

### Coupling between F_1_ and F_o_

In the complete complex, F_o_ and F_1_ are connected via two stalks: the central and the peripheral. The central stalk is the rotary shaft of F_o_F_1_ ATP synthase, composed of the rotor parts of F_o_ and F_1_ (Stock et al. 1999; Wächter et al. 2011). Together, the γε complex and the *c*-ring form the combined entity of the rotary shaft. F_o_ and F_1_ form the second stalk, termed the peripheral stalk. This is comprised of the δ subunit and the *b*_2_ dimer. The peripheral stalk is located on the lateral side of ATP synthase and firmly holds both of the stator parts of F_1_ and F_o_ from the outside, spanning from the bottom tip of F_1_ to the opposite side of the lipid bilayer (Rodgers and Capaldi 1998; Stock et al. 1999). In the ATP synthase, F_o_ and F_1_ exert rotational torque against each other (Junge et al. 1997; Noji and Yoshida 2001; Oster and Wang 2000). Under physiological conditions where *pmf* is sufficient and the rotational torque of F_o_ surpasses that of F_1_, F_o_ rotates the γ subunit in the reverse direction, a clockwise rotation viewed from the membrane side (from the top of Fig. 1).

The reverse rotation of F_1_ induces the reverse conformational change of the α_3_β_3_-ring, leading to the reverse reaction of ATP hydrolysis (ATP synthesis). When the free energy of ATP hydrolysis dominates, and the F_1_ generates a larger torque than F_o_, F_1_ reverses the rotation of F_o_ to induce active proton pumping and build up *pmf* across the membrane. Thus, F_o_ and F_1_ interchange the energy between *pmf* and free energy of ATP hydrolysis via the mechanical rotation of the rotor complex. For efficient energy interconversion, the central stalk and the peripheral stalk have to be resistant against mechanical torsional stress (Wächter et al. 2011). The δb_2_ subcomplex is known to form a stable complex (McLachlin et al. 1998). Regarding the central stalk, although the *c* subunit oligomer ring has multiple symmetric binding sites for the γε complex (Müller et al. 2004; Schulenberg et al. 1999; Watts et al. 1995), there have been, to date, no reports of slippage at the interface of the γε subcomplex and the *c*-ring. On the other hand, some studies suggest that the stalks are twisted in the ATP synthase at work. Although the elasticity of the peripheral stalk has been reported previously (Junge et al. 2009), recent studies suggest that the γ subunit of the central stalk is twisted (Okazaki and Hummer 2015; Vahidi et al. 2016). It remains elusive how the elasticity of the stalks contribute to energy transduction within the ATP synthase.

### H^+^/ATP stoichiometry

Since the stoichiometry of protons and ATP per rotation is not the same, the direction of the reaction, i.e., rotation is determined by the balance of total Δ*G*_ATP_ and *pmf* coupled with one turn of the γε–*c*-ring spindle:

$$ m\times \Delta {G}_{ATP}\ \mathrm{and}\  n\times pmf $$

where *m* and *n* represent the number of ATP and protons coupled with a single turn.

All of F_1_, of which crystal structures are available, show a pseudo three-fold symmetry in the αβ stator ring (Abrahams et al. 1994; Böttcher and Gräber 2000; Cingolani and Duncan 2011; Stocker et al. 2005). Thus, the stoichiometry of F_1_ (*m*) should be 3 ATPs per turn. Actually, all of the F_1_ tested in the rotation assay show stepping rotation with 120° intervals (Bilyard et al. 2013; Konno et al. 2006; McMillan et al. 2016; Suzuki et al. 2014; Yasuda et al. 1998). Thus, it is well established that *m* = 3.

On the other hand, the stoichiometry of proton translocation per turn (*n*) is variable between species, yet is invariant within species. It is assumed that *n* is determined by the number of *c* subunits in the oligomeric *c*-ring rotor of F_o_ (Junge et al. 1997). While the bacterial F_o_ has generally 10–15 *c* subunits in the oligomer ring (Ballhausen et al. 2009; Matthies et al. 2009; Meier et al. 2005a; Pogoryelov et al. 2005, 2009), mammalian mitochondrial F_o_ has 8 (Watt et al. 2010) and, depending on the origin, chloroplasts have 13–15 subunits in the *c*-ring (Pogoryelov et al. 2007). Several groups reported that F_o_F_1_ from *Escherichia coli* makes the rotation with 10 steps, which is consistent with the number of *c* subunits being 10 (Ballhausen et al. 2009; Düser et al. 2009; Ishmukhametov et al. 2010). It should be noted that the apparent number of rotation steps of F_o_F_1_ depends on the experimental conditions, because 10-step rotation should be observed only when the proton translocation or conformational change of F_o_ determines the overall rate of rotation. Otherwise, step rotation of F_o_F_1_ should represent the structural symmetry of F_1_, as previously reported (Watanabe et al. 2013).

Thus, the structural symmetry of the *c*-ring and α_3_β_3_-ring would set the stoichiometry ratio of protons against ATP as:

$$ {H}^{+}/ ATP= n/ m= n/3. $$

When we consider possible uncoupling rotation of F_o_, i.e., slipped rotation without accompanying proton translocation, this value sets the upper limit of the H^+^/ATP ratio. Before structural information on the *c*-ring became available, the H^+^/ATP ratio was considered to be an integer, such as 3 or 4, and to be common among species. Recent studies show that this is not the case. The H^+^/ATP ratio estimated from the structure is 2.7 for mammalian mitochondrial F_1_, 3.3 for *E. coli* F_o_F_1_, and 4 or more for chloroplast F_o_F_1_ (Ballhausen et al. 2009; Pogoryelov et al. 2005; Watt et al. 2010). Biochemical analysis supports that F_o_F_1_ with a larger number of the *c* subunit shows a larger H^+^/ATP ratio (Turina et al. 2016). One example is a comparative study on the H^+^/ATP ratio of F_o_F_1_ from *Saccharomyces cerevisiae* or *Spinacia oleracea* (Petersen et al. 2012), each of which has 10 or 14 *c* subunits (Symersky et al. 2012; Vollmar et al. 2009). The experimentally determined H^+^/ATP ratio was 2.9 and 3.9, respectively. The reason for the lower values than expected is unclear, although it could be attributable to the slipped rotation or a biased experimental error.

A higher H^+^/ATP ratio means that the F_o_ must exert higher torque to induce ATP synthesis under a given *pmf*. The difference in the H^+^/ATP ratio would represent different physiological requirements. When *pmf* is sufficiently high and stable over the life cycle of a cell, ATP production requires less protons (such as in mammalian mitochondria). However, when *pmf* is small and/or environmental ΔpH is inverted, as it happens in alkaliphilic extremophiles (McMillan et al. 2009) or chloroplasts (Pogoryelov et al. 2005), the F_o_ requires more protons to ensure constant ATP production.

The modulation of *c*-rings between organisms must be subtle, yet it is clearly essential, since it has been demonstrated, using mutagenesis, that a minor modification at the *c*–*c* interface can modulate the stoichiometry of the *c*-ring (Pogoryelov et al. 2012). The rotation-mediated energy coupling mechanism of F_o_F_1_ ATP synthase would ease the modulation of the H^+^/ATP ratio. This mechanism may be an advantageous feature to allow the adaptation of the ATP synthase for a variety of environmental conditions.

## Basic properties as a motor

### Rotation assays of the F_1_-ATPase

**Fig. 3:**
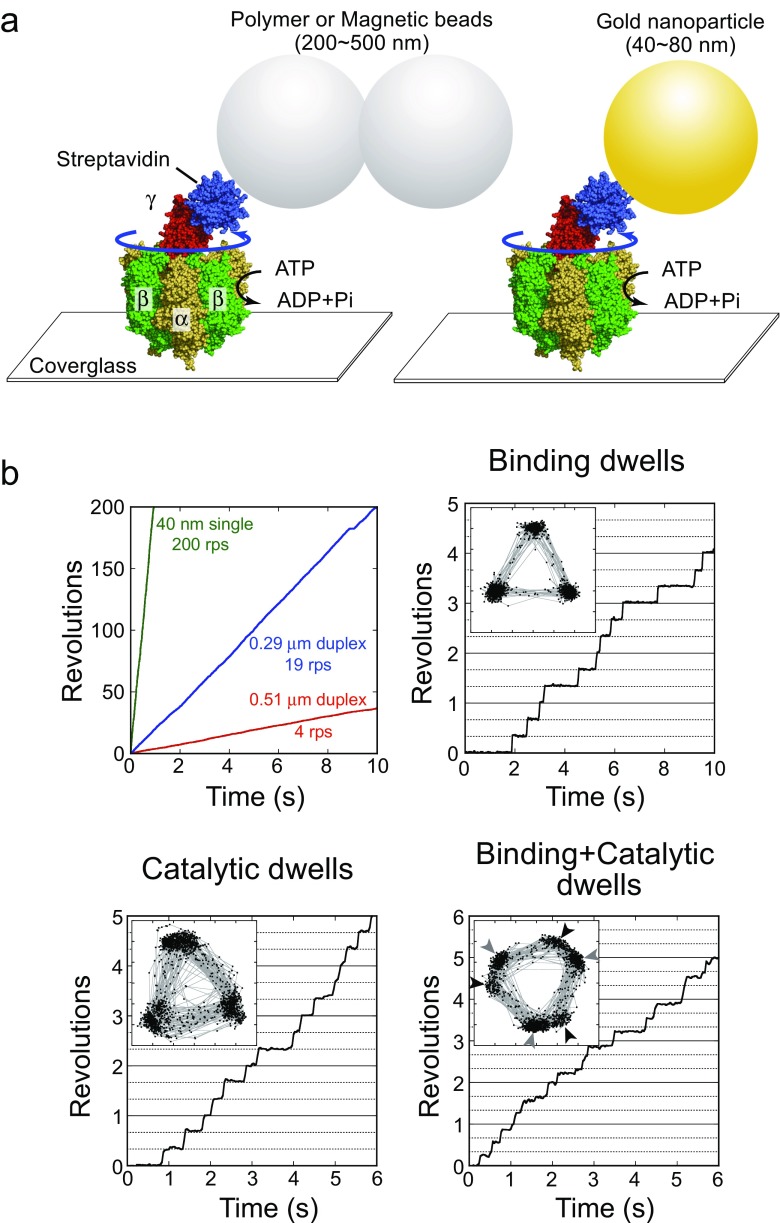
Rotation assay of F_1_. **a** A schematic image of the single-molecule rotation assay. The structural models of F_1_ (PDB ID: 1E79) (Gibbons et al. 2000) and streptavidin (PDB ID: 1N43) (Le Trong et al. 2003) are shown as sphere representation. The F_1_-ATPase α_3_β_3_-ring is immobilized on a glass surface, and an optical probe (fluorescently labeled actin filament, polymer beads, gold nanoparticle, gold nanorod, etc.) is attached to the γ subunit to visualize the rotary motion of γ subunit by an optical microscope. **b** Top left panel shows the time courses of rotation with various probe sizes under saturating ATP conditions (1∼5 mM ATP). The top right panel represents the time course of rotation of wild-type F_1_ under an ATP-limiting condition (60 nM ATP), where the dwell is caused by slow ATP binding. The inset shows the trajectory of the centroid of the optical probe. The bottom left panel shows the time course of rotation of a mutant F_1_ (βE190D) (Shimabukuro et al. 2003) under a saturating ATP condition (2 mM ATP). Each dwell is caused by the slow catalysis by the mutant F_1_. The bottom right panel shows the time course of rotation of a mutant F_1_ (βE190D) around the *K*_m_ region (2 μM ATP). In this condition, the 120° step splits into 0° and 80° substeps, each intervened with a binding dwell and catalytic dwell, respectively. The black and gray arrowheads indicate the positions of ATP binding and catalytic dwell, respectively

After the establishment of the rotation assay in 1997 (Noji et al. 1997), various types of methodologies for analysis of the rotation have been reported. In all protocols, the α_3_β_3_-ring of F_1_ is immobilized on the coverslip surface to suppress the rotary Brownian motion of the whole body of the F_1_ molecule. In most rotation assays, polyhistidine tags incorporated at the N terminus of the β or α subunit are used to anchor the α_3_β_3_-ring on the coverslip (Bilyard et al. 2013; Konno et al. 2006; McMillan et al. 2016; Suzuki et al. 2014; Yasuda et al. 1998). Then, a probe is attached onto the upwardly protruding part of the γ subunit for the visualization of the rotation of the γ subunit, of which the radius is far below optical resolution, having only a 1 nm radius (Abrahams et al. 1994). The first rotation assay employed a fluorescently labeled actin filament that was 0.5–5.0 μm in length (Noji et al. 1997). Due to the photobleaching effect of fluorescent dye that limits the observation duration, duplexes of submicron latex beads or magnetic beads that are observable in conventional bright field imaging are now preferentially used (Fig. 3a) (Hirono-Hara et al. 2001; McMillan et al. 2016; Yasuda et al. 2001). With submicron-sized probes, the viscous friction of a probe against fluid limits the maximum rotation velocity. A typical rotation rate with these probes under saturating ATP conditions has been reported to be between 5 and 20 revolution/s and readily identified by eye (Fig. 3b, top left).

Although viscous friction-limited conditions allow us to estimate the rotational torque individual molecules generate (see the section entitled “Torque”), detailed rotation dynamics such as stepping rotation and short-lived pausing states are frequently unclear due to the slow response of the probes used. For a detailed investigation of stepping rotation, nanoparticles of size several tens of nanometers, such as gold colloids or gold nanorods, are regarded as the most useful (Spetzler et al. 2006; Ueno et al. 2010; Yasuda et al. 2001). Nanoparticles do not behave like larger probes. Their response time is much faster, thereby enabling the observation of short-lived rotational pauses of F_1_. In the following section, the basic rotation properties of F_1_ revealed by rotation assay are discussed. Due to the breadth of knowledge available, we focus on the thermophilic *Bacillus* PS3 (TF_1_).

### Step rotation and reaction scheme

**Fig. 4:**
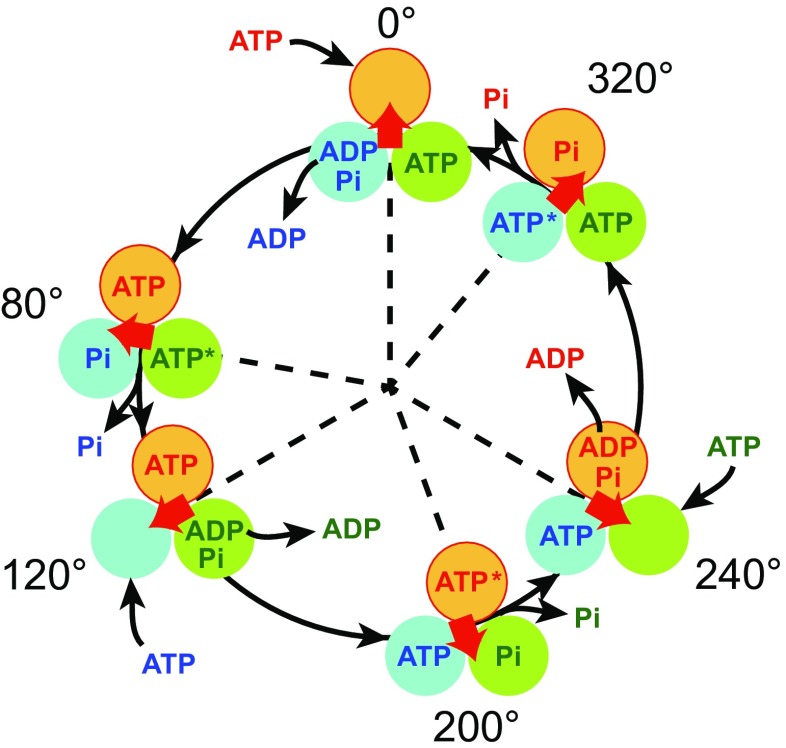
Proposed chemomechanical coupling scheme of TF_1_. Each *circle* represents the chemical state of the catalytic site in each β subunit. ATP* represents pre- or post-hydrolysis state of ATP. The *central red arrow* represents the orientation of the γ subunit. 0° is defined as the ATP binding angle for the catalytic site at the 12 o’clock position (*orange*). In this model, ATP bound at 0° is cleaved into ADP and Pi at 200°, ADP dissociates at 240°, and then phosphate release occurs at 320°. Other catalytic sites (*blue* and *green*) also obey the same reaction scheme offset by 120° and 240°

Rotation can be resolved into stepping rotation when the intervening pause durations dominate during the recorded period of rotation observation. Stepping rotation of F_1_-ATPase was first observed using an actin filament under low [ATP] conditions, where ATP binding is the rate-limiting step (Fig. 3b, top right). While ATP waiting time is controllable by changing medium [ATP], other catalytic states are not readily modulated. By achieving submillisecond imaging with gold colloid (*r* = 40 nm), a new rotation dwell with millisecond duration was found, revealing that the 120° step can be further resolved into 80° and 40° substeps (Yasuda et al. 2001). Kinetic analysis with a mutant F_1_ with slow catalysis revealed that the 80° and 40° substeps are initiated after ATP binding and hydrolysis of bound ATP, respectively (Shimabukuro et al. 2003). Thereby, the rotation dwell before each substep is referred to as binding, catalytic dwell, or pause. Several studies suggested that the ADP release occurs during binding dwell, and that the inorganic phosphate release takes place during catalytic dwell (Adachi et al. 2007; Nishizaka et al. 2004). Figure 4 shows the tentative reaction scheme of TF_1_. The exact timing of each reaction during pauses (at the end or at some midpoint) remains unclear.

Resolving mechanical motion into steps is a starting point to elucidate the chemomechanical coupling reactions scheme of molecular motors (Svoboda and Block 1994; Yildiz et al. 2003). Differing from architecturally simpler molecular motors like myosin and kinesin, F_1_ has three reaction centers. Naturally, this results in a comparatively more challenging puzzle to solve the reaction scheme. One may understand the difficulty when considering at which angle ATP is hydrolyzed after binding. It is important to note that the F_1_ has three catalytic angles in one turn and, thereby, there are, principally, three possible angles for each ATP to be hydrolyzed. A review paper that concisely introduces how this difficult puzzle has been addressed is available elsewhere (Okuno et al. 2011). Each reaction center conducts a single ATP hydrolysis event per 360° γ rotation, while the reaction phase of a catalytic site is always different from each other by 120°. Here, it should be stated that there is an unsettled argument on the timing of inorganic phosphate release. We propose that it occurs at 320°, i.e., during the third catalytic pause after binding (Watanabe et al. 2010), while Kinosita’s group support a 200° model (Adachi et al. 2007; Shimo-Kon et al. 2010). We are gaining strong supporting evidences for a 320° model from theoretical studies and structural analysis of MF_1_ (Rees et al. 2012); however, it remains to be resolved in TF_1_. We are certain further discussion and analysis will resolve this curious facet.

Interestingly, the rotation assay on mammalian F_1_ (human mitochondria F_1_; hMF_1_) showed that the 120° step is resolved into three substep positions at 65°, 25°, and 30° (Suzuki et al. 2014). Although the reaction scheme of MF_1_ apparently differs from that of TF_1_, it can be considered to be a variation of the reaction scheme of TF_1_, where the hydrolysis dwell and inorganic phosphate release dwell are split into different angles. Interestingly, a recent intensive analysis using data mining techniques revealed that TF_1_ makes a small rotational movement during the catalytic pause that had not been previously resolved using conventional data analysis (Li et al. 2015). Thus, the reaction scheme seems likely to be conserved among species, although there are some variations in substep size and sequence of reaction that might reflect physiological requirements. Regarding this point, a theoretical study suggests that the position of the substeps is not critical for energy coupling efficiency (Mukherjee and Warshel 2015).

### Torque

The motion of micron-sized objects (Brownian particles) in fluid is overdamped. The acceleration and deceleration time for Brownian particles is extremely short, meaning that the Brownian motion can be treated as a Markov process. When the F_1_ rotates a probe in a buffered solution, the viscous friction against the rotating probe is always balanced with the torque that the F_1_ generates. Thus, the torque of the F_1_ can be estimated from the viscous friction determined from the probe size (i.e., the radius for a spherical probe) and observed angular velocity (Noji et al. 2001; Pänke et al. 2001; Yasuda et al. 1998). The torque of TF_1_ has been repeatedly reported to be around 40 pNnm (Hayashi et al. 2010; McMillan et al. 2016; Noji et al. 2001; Ueno et al. 2014). In addition, the angular velocity profile is almost constant against rotational angle. Thus, TF_1_ is generally considered to generate a constant 40 pNnm of torque, irrespective of rotational angle.

One possible concern about this torque estimation is that the torque could be underestimated. This is because the viscosity of fluid near the surface is known to be higher than that in the middle. On the other hand, the surface effect could be minor, because a probe is lifted by at least 20 nm perpendicular to the coverslip surface by the molecules involved in the rotation assay (F_1_, biotin, streptavidin, and polymer cushion on the probe surface). Furthermore, rotating particles have a slight elevation angle, being apart from the surface at the rotation edge of the probe where viscous friction is dominant. This concern was addressed, at least partially, by application of a statistical physical theory known as the fluctuation theorem. A variation of the fluctuation theorem formula adjusted to the rotation assay parameters allows for an estimation of entropy generation, i.e., the force generation only from the trajectory of probes without the consideration of physical parameters requisite for the previous method, such as fluid viscosity or size or shape of probes. A sole, but critical, requirement of this analytical method is the rapid and precise recording of the probe position. This method reconfirmed that TF_1_ generates a torque of 40 pNnm (Hayashi et al. 2010).

The torque of 40 pNnm has important implications for the energy coupling of TF_1_. Torque multiplied by angular displacement gives the work done by the molecular motor. In the case of the F_1_, the estimated work that the F_1_ does per 120° rotational movement (=single turnover of ATP hydrolysis) is approximately 80 pNnm (Yasuda et al. 1998). This is consistent with the free energy of ATP hydrolysis in a cell where Δ*G’*_ATP_ is 48 kJ/mol, which is equivalent to 80 pNnm/molecule. In addition, theoretical studies suggest that a constant torque over rotation angle is also reasonable for efficient energy coupling (Oster et al. 2000). However, recent reports challenge this view, which invite revision of the current understanding of the torque profile. Saita et al. (2015) reported that the torque profile showed three sawtooth-like peaks in a 120° step. Because the observed profile is not consistent with any reported substep positions, a reasonable explanation for this conflict with previous reports is yet to be described. Sielaff (2016) reported a different torque profile. They observed that the instantaneous angular velocity of rotation probed with a gold nanorod showed a distinct peak during a 120° step. Because the nanorod has a low viscous drag coefficient, it is not clear if the observed angular velocity represents the genuine torque profile of the F_1_ or it includes some effect from intermolecular friction between the rotor and the stator of the F_1_.

We should note here that the torque could be different among species. Another model F_1_ well studied with rotation assay is the F_1_ from *Escherichia coli* (EF_1_). Although several groups have reported different values, the reported torque of EF_1_ ranges from 30 to 63 pNnm (Bilyard et al. 2013; Hornung et al. 2008; Pänke et al. 2001). Recently, an F_1_ from a thermoalkaliphilic bacterium, *Caldalkalibacillus thermarum*, was reported to generate torque of over 50 pNnm (McMillan et al. 2016). The V_1_ motor of V-type ATPase from bacteria is also well studied using the rotation assay. There is a clear trend that V_1_-ATPases generate a lower torque than F_1_-ATPases: the V_1_ from *Thermus thermophilus* generates 30–35 pNnm and the Na^+^-transporting V-ATPase generates 23 pNnm of torque, respectively (Hayashi et al. 2010; Imamura et al. 2005; Ueno et al. 2014). Although more data are required, we can see a tendency that F_1_ or V_1_ dedicated only to ATP synthesis in a cell generates a larger torque than motors working as ATP-driven ion pumps (McMillan et al. 2016).

### Torque-generation step

A milestone to meet in elucidating the mechanochemical coupling of molecular motor proteins is to identify which reaction step is principally responsible for force generation. ATP hydrolysis is resolved into at least four elementary steps: ATP binding, hydrolysis, ADP release, and inorganic phosphate release. This is a technically challenging question to address. Force measurement is a straightforward strategy. However, the catalytic reactions following ATP binding are often too fast to be resolved using force measurement as a sole strategy. Allostericity among multiple reaction sites on a motor protein also hampers the identification of the force-generation step. Another strategy that gives clues to this process is structural analysis. Interestingly, after nucleotide binding, large conformational rearrangements have been observed in the crystal structures of motor proteins.

The crystal structures of the F_1_ have revealed distinct conformational difference between the β subunits with and without bound nucleotide (Fig. 2) (Cingolani and Duncan 2011; Duncan et al. 1986; Ferguson et al. 2016; Menz et al. 2001). Several crystal structures of F_1_-ATPases prepared in different conditions and with different ligands have been reported so far (Abrahams et al. 1996; Braig et al. 2000; Cabezón et al. 2003; Kagawa et al. 2004; van Raaij et al. 1996). The β subunits are the principal torque generators, and assume two distinct conformations in most structures. The β subunit without bound nucleotide is in open conformation, while the β subunit with bound nucleotide takes closed conformation, in which the C-terminal domain swings toward the γ subunit (Fig. 2b) (Abrahams et al. 1994). From this observation, the ATP-binding step is proposed to be a major torque-generating step. This is quite consistent with the observation that the substep size induced by ATP binding is two times larger than that initiated after hydrolysis and inorganic phosphate release (Yasuda et al. 2001).

An alternative approach to study the force-generating step is to investigate how rotation modulates the equilibrium constant of individual reaction steps, *K*_E_^reaction^. Standard free energy/molecule is derived from *K*_E_^reaction^ as follows:

$$ \Delta {G}^{\mathrm{o}'}={k}_{\mathrm{B}}\mathrm{T}\cdot \ln {K_{\mathrm{E}}}^{\mathrm{reaction}} $$

where *k*_B_T represents thermal energy. Then, free energy change upon rotation, $\frac{\partial G}{\partial \theta}$ represents the magnitude of the energy that is released upon the reaction. Thus, the angle-dependent modulation of *K*_E_^reaction^ is a good barometer to estimate torque generation. For this purpose, the rotation angle of the probe (a magnetic bead duplex) was controlled with magnetic tweezers, and the reaction probability was investigated as a function of rotation angle (Watanabe et al. 2012). This work revealed that TF_1_ exponentially tightens the affinity to ATP with rotation, while the equilibrium constant of ATP hydrolysis is slightly shifted toward hydrolysis direction. The estimated energy generation is 21–54 pNnm for affinity change of ATP and 4–17 pNnm for equilibrium shift of hydrolysis. The reason for the range of values is that the γ subunit must be twisted to some extent during manipulation, and the actual orientation of the γ subunit may be overestimated. The upper and lower limits represent the estimated energy change with or without considering the elasticity of the γ subunit. Although the exact quantification of energy release has not yet been done, this study showed that the contribution of affinity change of ATP for torque generation is over 2-fold larger than that of ATP hydrolysis.

Thus, many lines of experimental results have shown that the ATP-binding process, or more precisely the affinity change to bound ATP, is a major torque-generation step in F_1_ catalysis, while the chemical cleavage of ATP has relatively minor contribution. It is less well understood how torque is generated upon ADP and inorganic phosphate release. Classic biochemical studies have shown that the inorganic phosphate uptake step is an energy-requiring step during ATP synthesis, suggesting that the inorganic phosphate-releasing step is another torque-generation step. This idea was supported by kinetic analysis on rotation of F_1_ in the presence of excess inorganic phosphate (Adachi et al. 2007).

## Robustness

### Robustness of torque transmission

**Fig. 5:**
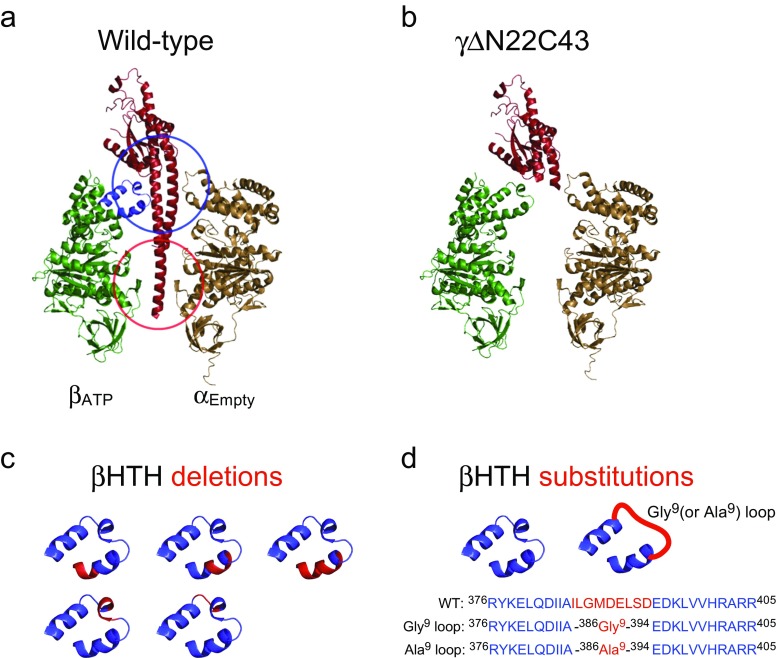
Stator–rotor interactions within F_1_. **a** α_Empty_–β_ATP_ pair and the γ subunit are shown as cartoon representation (PDB ID: 1E79). The *blue and red circles* indicate the orifice and sleeve regions in the α_3_β_3_-ring, respectively, at which the γ subunit is held. The helix-turn-helix (HTH) structure of the β subunit forming the main interface of the stator orifice is colored *blue*. **b** The structure of axle-less mutant γΔN22C43 (Furuike et al. 2008). The 22 residues of the N-terminal helix and the 43 residues of the C-terminal helix in the γ subunit are deleted. **c** The positions of deletion in the HTH structure are shown in *red* (Usukura et al. 2012). **d** All residues of the HTH in contact with the γ subunit are substituted with glycine or alanine (*red region*) (Tanigawara et al. 2012)

Structural mapping to explore which stator–rotor interaction is the principal method for the understanding of torque transmission. The interactions revealed by this approach have provided major insights for the elucidation of chemomechanical coupling of F_1_. The γ subunit is composed of a globular domain with an α/β fold and an antiparallel coiled-coil domain of the N- and C-terminal helices. As shown in Fig. 5a, the γ subunit is held in the α_3_β_3_ stator ring in two positions: the upper orifice, from which the globular domain of the γ subunit protrudes vertically, while the lower hydrophobic sleeve holds the tip of the C-terminal helix.

The stator–rotor interactions have been actively studied since 1994, when the crystal structure of F_1_ became available (Abrahams et al. 1994) and many residues that impair catalysis were identified. Subsequently, the rotation assay was established, and some of these mutants were closely examined. No significant impact on the torque generation was observed using these mutants; however, kinetic parameters were affected to some extent (Hara et al. 2000; Omote et al. 1999). The robustness of the torque transmission between stator and rotor was more clearly shown by Kinosita’s group. They showed that the F_1_ still rotates even after being devoid of the entire γ subunit coiled-coil axle (Fig. 5b) (Furuike et al. 2008). Although the rotation rate of 1 Hz was much slower than that of the wild-type F_1_ (∼200 Hz) and the torque was too low to measure, the rotation was apparently unidirectional. This finding came as a big surprise. This result means that, for the most part, the stator–rotor interaction is dispensable for unidirectional rotation. The remaining interaction in the axle-less F_1_ was the upper half of the orifice interaction.

Following this, the orifice interaction for torque transmission became the focus of the following study. The helix-turn-helix (HTH) structure of the β subunits forming the main interface of the stator orifice was investigated (Usukura et al. 2012). Figure 5a highlights the HTH structure in blue. The HTH structure is a part of the C-terminal domain of the β subunit, which makes a large swing motion upon nucleotide binding (Abrahams et al. 1994; Masaike et al. 2008). The HTH structure was shortened by the deletion of one or two turns (Fig. 5c). The resultant mutant F_1_ still showed clear rotation (Usukura et al. 2012). The orifice interaction was comprehensively shown to be dispensable. This point was confirmed in another mutagenesis study where all residues of the HTH in contact with the γ subunit were substituted with alanine or glycine residues (Fig. 5d) (Tanigawara et al. 2012). Surprisingly, the mutant F_1_ with Ala substitutions generated a torque comparable with the wild-type F_1_ values (97% of the wild-type), suggesting that specific interactions at the orifice interface are not entirely requisite for full torque transmission. The mutant F_1_ with Gly substitutions replacing 22 residues of the HTH still rotated; however, the torque was reduced to 60% of the wild-type. Molecular dynamics simulations suggested that the Gly mutant completely lost the rigidity of the HTH structure, while the Ala mutant still keeps the structure of the HTH (Tanigawara et al. 2012). These findings suggest that full torque transmission at the orifice interface does not require specific interaction, but the rigidity of the HTH structure. It is highly likely that the HTH structure works as a “push rod” for the β subunit to induce unidirectional rotation.

Thus, the series of extensive mutation studies revealed that all parts of the stator–rotor interface are dispensable, and that the torque transmission mechanism is far more robust than previously thought. The mutagenesis studies suggest that the orifice interaction is responsible for roughly half of the torque transmission. The sleeve interaction probably transmits the other half of the torque. The remaining issue to be explored is how the α_3_β_3_-ring generates torque at the sleeve interaction. From the crystal structures, it is not clear what type of conformational change is responsible for the torque transmission at the sleeve interaction.

### Robustness of allostericity: from dictator model to professor model

The robustness of torque transmission also implies the robustness of the cooperativity among the three catalytic reaction centers. The rotation of axles-less F_1_ brought surprise not only in the context of the torque transmission, but also in that of the allostericity of F_1_. This is because, at the time, the so-called γ-dictator model was prevailing. This model assumes that the interaction with the γ subunit fully controls the timing of catalysis in the β subunits. Another underlying reason is a biochemical study which showed that the isolated α_3_β_3_-ring did not show obvious cooperativity among catalytic sites (Kaibara et al. 1996). The following experiments that reinforced the γ-dictator model were demonstrations that ATP synthesis is possible upon forced reverse rotation of F_1_ (Itoh et al. 2004; Rondelez et al. 2005). These experiments showed that the equilibrium constant of ATP hydrolysis is over 10^5^ at room temperature in aqueous solution, and can be easily modulated to be less than 1 by controlling the angle orientation of the γ subunit.

**Fig. 6:**
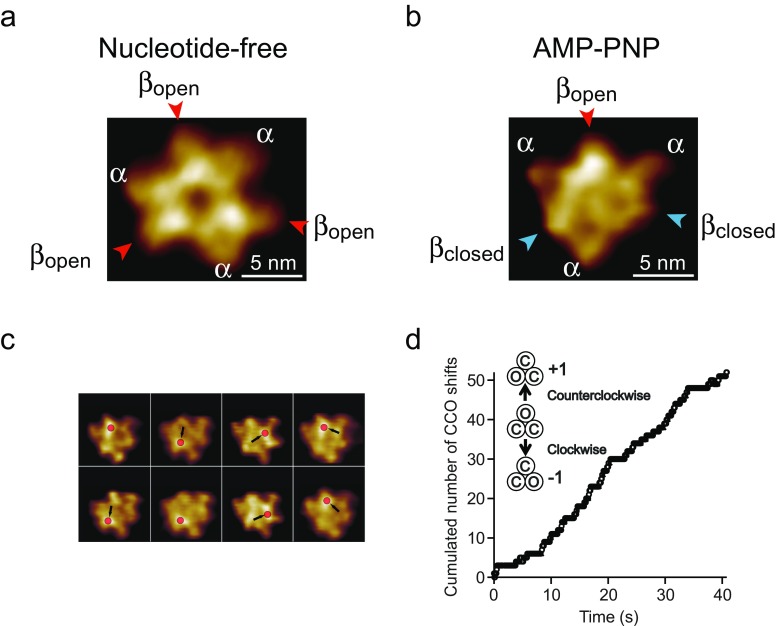
High-speed atomic force microscopy (HS-AFM) imaging of the isolated α_3_β_3_-ring. **a** Averaged AFM image without nucleotide when observed from the C-terminal side. The *red arrows* indicate β subunits showing an open conformation (Uchihashi et al. 2011). **b** Averaged AFM image at 1 mM AMP-PNP. The *blue arrows* indicate the β subunits showing a closed conformation. **c** Successive HS-AFM images showing conformational change of the β subunits at 2 μM ATP (left-to-right and top-to-bottom). The *red circles* indicate the highest pixel in each image. The frame rate of imaging is 12.5/s. **d** Time course of the cumulated number of counterclockwise shifts of the CCO state. The *black circles*, *crosses*, and *pluses* represent CCO, COO, and other irregular states, respectively

The aforementioned mutagenesis studies on the stator–rotor interface imply that there is no indispensable stator–rotor interaction to control cooperative catalysis for unidirectional rotation. To visualize sequential catalysis accompanying the power-stroke motion of the β subunit in the isolated stator complex (the α_3_β_3_ subcomplex), high-speed atomic force microscopy (HS-AFM) was employed (Fig. 6). This imaging method allowed visualization of the structural change of biomolecules at work in aqueous solution at over 10 frames per second with subnanometer spatial resolution (Kodera and Ando 2014; Uchihashi et al. 2011). The isolated α_3_β_3_-ring was covalently immobilized on mica surface for HS-AFM observation. In agreement with the crystal structures of F_1_, when the unhydrolyzable nucleotide (adenylyl-imidodiphosphate, AMP-PNP) was added to the solution, the α_3_β_3_-ring adopts a CCO state. Two of the three β subunits take the closed form (C), where the C-terminal domain swings down, while the third took the open form (O), with the C-terminal domain protruding vertically (Fig. 6b). Dynamics were also observed in the presence of ATP. The α_3_β_3_-ring rotationally propagated the CCO state in a counterclockwise direction similar to the γ rotation; when the β in the C state at the clockwise side of a CC pair makes a conformational C-to-O transition, the β in the O state makes an O-to-C transition (Fig. 6c, d). The rate of the transition (1.5 s^−1^ at 2 μM ATP) was consistent with the rate of ATP hydrolysis measured in biochemical analysis (1.6 s^−1^ at 2 μM ATP). Thus, it was clearly shown that three β subunits undergo sequential power-stroke conformational transitions coupled with ATP hydrolysis.

This is the decisive evidence that the α_3_β_3_-ring has intrinsic cooperativity among the three catalytic sites, and that the γ subunit dictator model is likely incorrect for F_1_. However, the rate of the observed conformational transition, 1–4 Hz, was remarkably slower than with the γ subunit, being 100–200 Hz. In addition, the occasional back-step was also observed, suggesting that the cooperativity does not reach perfection without the γ subunit. Therefore, we propose that the “professor” model might be a better representation for the allostery of F_1_ than “intrinsic cooperativity”. Here, “professor” means an existence that is not indispensable but enhances the activity of a system or organization.^1^

**Fig. 7:**
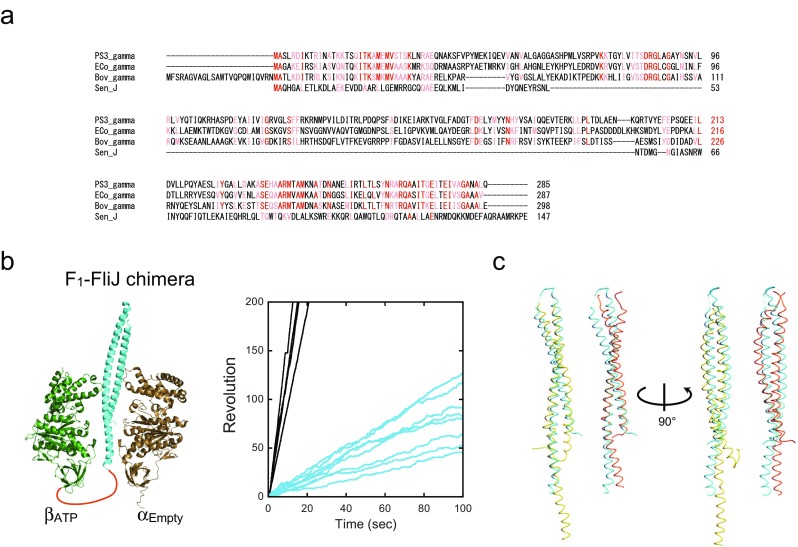
Rotation of F_1_-FliJ chimera. **a** Sequence alignment of FliJ and F_1_-γ subunit. The amino acid sequences of F_1_-γ from thermophilic *Bacillus* PS3 (PS3-γ), *Escherichia coli* (Eco-γ), and bovine mitochondria (Bov-γ) were aligned using ClustalW. The FliJ sequence (Sen-J, PDB ID: 3AJW) and Bov-γ (PDB ID: 1E79) were structurally aligned using the MATRAS server due to the low sequence similarity between them (Baba et al. 2016). The conserved residues are highlighted in *red* (identical) or *pink* (strong similarity). **b** Left panel shows the structure model of F_1_-FliJ chimera. α_Empty_–β_ATP_ pair (PDB ID: 1E79) and FliJ (*cyan*, PDB ID: 3AJW) are shown as cartoon representations. The linker portion is represented by an *orange line*. The right panel shows the time courses of rotation of F_1_ (*black lines*) and F_1_-FliJ chimera (*cyan lines*). **c** Structural alignment of FliJ and V_1_-D or F_1_-γ subunit. FliJ (*cyan*, PDB ID: 3AJW) was superimposed on the D subunit of V_1_ (*yellow*, PDB ID: 3W3A) or the γ subunit of F_1_ (*orange*, PDB ID: 4XD7) using the MATRAS server. Root-mean-square deviations (RMSDs) between FliJ and the D subunit of V_1_ or γ subunit of F_1_ are 3.2 Å or 4.4 Å, respectively

A question that then arose based on this finding was “can an exogenous rod-shaped protein rotate in the α_3_β_3_-ring?” To test this, attempts were then made to insert the FliJ protein into the α_3_β_3_-ring (Fig. 7) (Baba et al. 2016). FliJ is a component of the bacterial flagellar type III export system and its function has not yet been described (Ibuki et al. 2011). FliJ also has an antiparallel coiled-coil with a similar length to the γ subunit; therefore, it has a similar morphology. Importantly, there is no homology in the primary structure between FliJ and the γ subunit (Fig. 7a), and the surface charge density is opposite to each other. FliJ has a net negative charge, while the γ subunit has more positively charged residues. The resultant hybrid motor showed unidirectional rotation in the counterclockwise direction (Fig. 7b, right, cyan lines). However, torque was only 10% of the torque of the wild-type F_1_ (Baba et al. 2016). FliJ was also inserted into the stator ring from a V-ATPase to ensure that the robustness of torque transmission is conserved between F_1_ and V_1_ molecular motors. Surprisingly, the V_1_-FliJ hybrid motor showed active unidirectional rotation with a measured torque that is comparable to that of the native V-ATPase (Baba et al. 2016). This is probably because the shape of the rotor from the V_1_-ATPase is more similar to FliJ than the γ subunit of F_1_ (Fig. 7c). These findings strongly suggest that the torque transmission in F- and V-type ATPases do not require any residue-specific interaction between stator and rotor, but do require gross special/shape matching for efficient torque transmission.

### Robustness of catalytic power

Robustness was also revealed in the catalytic power of the F_1_. Although the F_1_ is unique in its high reversibility and efficiency of the chemomechanical coupling, the F_1_ shares common structural features of the catalytic site with other nucleotide-driven molecular machines. One of the most well conserved structural features is the phosphate-binding motif, the so-called p-loop, or the Walker motif B that is found in most NTPases. The p-loop motif has a GXXXXGKT/S sequence (Walker et al. 1982). The seventh lysine, the p-loop lysine, is the most conserved, and is the gold standard target to knock out the catalytic activity of any described NTPase. In support of this, when substituted with another non-charged residue such as alanine, the catalytic activity reduces to undetectable levels in biochemical analysis (Senior and Al-Shawi 1992; Yagi et al. 2009). Substitution of the p-loop lysine in F_1_ is known to abolish all ATPase activity (Hanson and Whiteheart 2005).

The F_1_-ATPase has another well-conserved charged residue directly associating with bound ATP. Many of the NTPase proteins share a glutamic acid residue that seems to associate with bound nucleotide via a coordinated water molecule at the distal end of the γ-phosphate. This glutamic acid residue is critically important for the catalysis of F_1_ (Shimabukuro et al. 2003). When substituted with an aspartic acid, the rate of F_1_ catalysis is reduced by over 100-fold. When substituted with non-charged residue, the catalytic activity was undetectable in biochemical assays (Amano et al. 1994, 1999). Previously, it was proposed that the glutamic acid residue activates the coordinated water by removing a proton and induces the in-line attachment of the activated water to the γ-phosphate of ATP. Therefore, the glutamic acid residue was termed a “general base”. However, a recent theoretical study revised the working mechanism of the glutamic acid residue, showing that hydrolysis at the γ-phosphate spontaneously occurs before the protonation of the glutamic acid residue (Hayashi et al. 2012). The proposed role of this residue is to build a hydrogen network for the enhancement of proton transfer, the kinetically rate-determining step of ATP hydrolysis.

Another common feature of the catalytic site of F_1_ with other NTPase proteins is the catalytic arginine residue, the so-called arginine finger. The arginine finger was first found in a G-protein activating protein (GAP), and was proposed to be a catalytic switch to initiate the hydrolysis of GTP bound on G-protein (Ahmadian et al. 1997). The corresponding arginine residues were widely found in other NTPases, including RecA type proteins, of which F_1_ is a more distal family member, and to the AAA^+^ family proteins (Hanson and Whiteheart 2005). The arginine finger of F_1_ resides on the α subunit. Many lines of experimental research have shown that the arginine finger has a crucial role in the catalysis of F_1_ (Hatch et al. 1995; Komoriya et al. 2012).

These three charged residues of F_1_ were identified as catalytically critical residues in theoretical studies (Dittrich et al. 2004). Mutagenesis studies also showed that, when these residues are substituted with non-charged residues such as alanine, the catalytic power is abolished (Senior and Al-Shawi 1992; Yagi et al. 2009). Alanine mutants at these residues were re-investigated in rotation assay systems to confirm their critical role in catalysis (Watanabe et al. 2014). Against all expectations, all of the mutants showed unidirectional rotation. The rotational rate was significantly lower by a factor of 10^3^ to 10^4^. Such a low catalysis rate is not detectable in biochemical analysis. While the large impact of the mutation on catalysis is consistent with previous reports and theoretical analysis, the robustness of the catalytic power is unexpected. This finding reveals that the catalytic site architecture is designed to be quite robust against both point mutations and possibly more severe mutagenic perturbations.

Interestingly, all of the alanine mutants had a lower torque than the wild-type enzyme. The most remarkable impact was found in the alanine substitution at p-loop lysine, causing a 75% reduction of torque. Arginine finger and catalytic glutamic acid residue mutants also resulted in a torque reduction of 50%. This is in contrast to the impact of chemical modification of ATP on torque generation (Arai et al. 2014). The F_1_ has broad nucleotide specificity, hydrolyzing other nucleotides: GTP, CTP, and UTP, albeit with changes in the catalysis rate. Even when the base structure is removed, the F_1_ hydrolyzes base-free nucleotide, inducing γ subunit rotation. The impact of the modification or depletion of base in kinetics was remarkable; the binding constants of UTP and base-free nucleotide were 10^3^ and 10^6^ times slower than ATP. However, rotational torque was retained at a comparable level to that of ATP-driven rotation. This means that the interaction with the base structure of nucleotide is critical for the enhancement of binding, but not for torque generation. This is in contrast to the impact of mutation at the phosphate-binding residues, which largely impaired the kinetic power as well as torque generation (Arai et al. 2014; Watanabe et al. 2014).

**Fig. 8:**
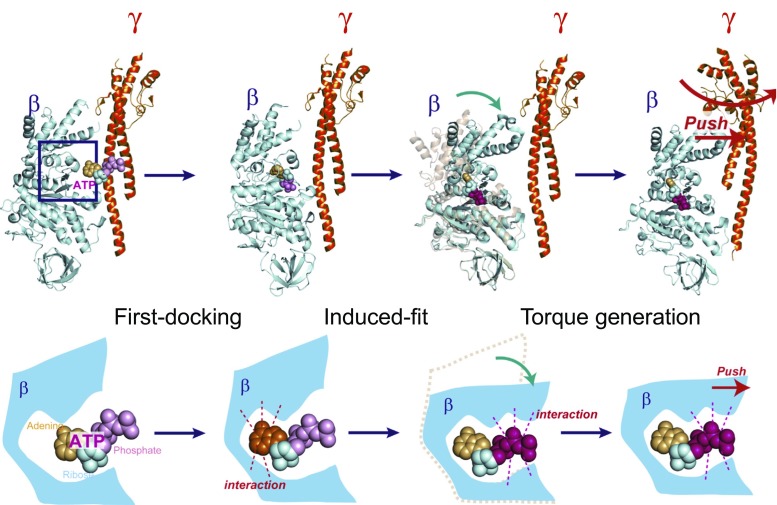
Two-step ATP binding model. The upper and lower panels show the conformational states of the β subunit and the chemical states in the binding pocket during ATP binding and subsequent torque generation. The ATP-binding process consists of two steps (first docking and second induced fit). The first docking process is triggered by the recognition of the base portion of ATP, and the subsequent induced-fit process is triggered by the recognition of the phosphate portion, which contributes to the torque generation (Arai et al. 2014)

As previously mentioned, the affinity change to bound ATP is a major torque-generation step. Considering these findings, we propose a two-step ATP binding model: the first substrate docking process and the following induced-fit process accompanying affinity change. The base structure of ATP is responsible for the first substrate recognition process, but it is not involved in the induced-fit process, while the binding of phosphate to the catalytic residues is principally responsible for the induced-fit process. A schematic view is depicted in Fig. 8. Considering the common structural features in the catalytic site, the phosphate-induced power-stroke mechanism is highly likely to be conserved among other molecular machines.

## Perspective

During the past two decades, single-molecule rotation assays have uncovered many aspects of the F_1_-ATPase catalytic mechanism. Basic properties of the F_1_ as a rotary motor, such as step size, rotational velocity, torque, and kinetic parameters of rotation, were all clearly revealed. Most parts of the reaction scheme have also been elucidated in combination with knowledge from structural analysis and biochemical studies. Theoretical studies have also been exceedingly helpful in contributing to the elucidation of the molecular mechanism of the F_1_. The torque-generation mechanism is also partly revealed. However, one must note that analysis and interpretation do not guarantee our understanding. A practical test would be an engineering approach where we design, build, and test chimeras of molecular motors. A hybrid motor of F_1_ and FliJ is an initial step toward the engineering approach (Pogoryelov et al. 2012), but we are still clearly at the preliminary stages of this approach. Theoretical toolboxes for de novo designing of proteins are growing rapidly. By combining the rational designing methods with directed evolution technology, we will soon be able to build de novo molecular motors. The feedback loop between analytical approaches represented by single-molecule rotation assays, and structural analysis, and engineering approaches will further our understanding of mechanochemical coupling mechanisms and design principles in naturally occurring protein machinery.

## Footnotes

1 Of course, this is a self-deprecating analogy. It could only be the case in Japan.
